# Dual-Functional AgNPs/Magnetic Coal Fly Ash Composite for Wastewater Disinfection and Azo Dye Removal

**DOI:** 10.3390/molecules30153155

**Published:** 2025-07-28

**Authors:** Lei Gong, Jiaxin Li, Rui Jin, Menghao Li, Jiajie Peng, Jie Zhu

**Affiliations:** 1 National-Local Joint Engineering Research Center of Biomass Refining and High-Quality Utilization, Institute of Urban & Rural Mining, Changzhou University, Changzhou 213164, China; 2 School of Petrochemical Engineering, Changzhou University, Changzhou 213164, China; 3 School of Environment Science and Engineering, Changzhou University, Changzhou 213164, China

**Keywords:** nano-silver composite, magnetized coal fly ash, antibacterial activity, methyl orange degradation, sustainable wastewater treatment

## Abstract

In this study, we report the development of a novel magnetized coal fly ash-supported nano-silver composite (AgNPs/MCFA) for dual-functional applications in wastewater treatment: the efficient degradation of methyl orange (MO) dye and broad-spectrum antibacterial activity. The composite was synthesized via a facile impregnation–reduction–sintering route, utilizing sodium citrate as both a reducing and stabilizing agent. The AgNPs/MCFA composite was systematically characterized through multiple analytical techniques, including Fourier transform infrared spectroscopy (FTIR), scanning electron microscopy (SEM), transmission electron microscopy (TEM), X-ray diffraction (XRD), and vibrating sample magnetometry (VSM). The results confirmed the uniform dispersion of AgNPs (average size: 13.97 nm) on the MCFA matrix, where the formation of chemical bonds (Ag-O-Si) contributed to the enhanced stability of the material. Under optimized conditions (0.5 g·L^−1^ AgNO_3_, 250 °C sintering temperature, and 2 h sintering time), AgNPs/MCFA exhibited an exceptional catalytic performance, achieving 99.89% MO degradation within 15 min (pseudo-first-order rate constant *k_a_* = 0.3133 min^−1^) in the presence of NaBH_4_. The composite also demonstrated potent antibacterial efficacy against *Escherichia coli* (MIC = 0.5 mg·mL^−1^) and *Staphylococcus aureus* (MIC = 2 mg·mL^−1^), attributed to membrane disruption, intracellular content leakage, and reactive oxygen species generation. Remarkably, AgNPs/MCFA retained >90% catalytic and antibacterial efficiency after five reuse cycles, enabled by its magnetic recoverability. By repurposing industrial waste (coal fly ash) as a low-cost carrier, this work provides a sustainable strategy to mitigate nanoparticle aggregation and environmental risks while enhancing multifunctional performance in water remediation.

## 1. Introduction

The sustainable development of human society is increasingly challenged by persistent global pollution, with industrial effluent discharge emerging as a critical contributor. Among various industrial contaminants, textile printing and dyeing wastewater stands out for its formidable treatment challenges, characterized by a high volume, elevated organic pollutant concentrations, complex chemical composition, and significant fluctuations in water quality parameters [1]. Azo dyes, such as methyl orange (MO) and its derivatives, are especially problematic due to their intricate molecular structures and exceptional stability, which render them highly resistant to microbial degradation and enable their long-term persistence in ecosystems [2]. Alarmingly, these recalcitrant compounds can undergo secondary transformations into more complex derivatives, potentially augmenting their mutagenic and carcinogenic properties, thereby posing severe risks to ecological balance and human health [3].

Current strategies for the removal of dyes include adsorption, reverse osmosis, membrane separation, and chemical oxidation. Chen et al. [4] developed a new type of nanocomposite membrane by embedding graphene oxide membranes (GO) and ZIF-8 nanoparticles into each other. Compared with the original GO reference membrane, the prepared GO/ZIF-8 (GZ) nanocomposite membrane (i.e., GZ membrane) had twice the permeability and the separation performance was also improved. In addition, the membrane also had good anti-fouling performance, and the removal effect on salts and organic pollutants was relatively ideal. Wang et al. [5] synthesized flower-like MoS_2_/g-C_3_N_4_ nanocomposites with a size range of 200–400 nm and a specific surface area of 70.656 m^2^/g as new methylene blue (MB) adsorbent materials. The results showed that the maximum adsorption capacity for MB reached 278.4 mg·g^−1^ at 45 °C. But these methods are hindered by substantial limitations, such as exorbitant operational costs, incomplete organic dye degradation, and the inadequate addressing of coexisting microbial hazards [6,7]. The heterogeneous and variable nature of industrial wastewater further fosters the proliferation of harmful microorganisms, including pathogenic strains, which exacerbate the risks to environmental and public health. These challenges underscore the urgent need for cost-effective, efficient, and multifunctional technologies capable of concurrently mitigating dye contamination and microbial threats in wastewater systems.

Nanoparticles, particularly silver nanoparticles (AgNPs), have attracted significant attention due to their distinctive physicochemical properties and versatile applications in antimicrobial therapy, catalysis, sensing, and materials science [8]. Their pronounced antimicrobial efficacy, stemming from the ability to disrupt microbial cell membranes and inhibit genetic material synthesis, offers a promising solution to combat antibiotic resistance and multidrug-resistant microbial strains prevalent in industrial and medical settings [9]. For instance, Singh et al. [10] reported the dose-dependent antimicrobial activity of AgNPs, achieving substantial growth inhibition of bacteria at concentrations between 0.5 and 2 mg·mL^−1^, highlighting their therapeutic potential in managing bacterial infections. Korkmaz et al. [11] further reported that AgNPs synthesized using *Ficus carica* leaf extract exhibited potent antibacterial and anticancer activities, underscoring their biocompatible and multifunctional nature. Eslam et al. [12] showed that AgNPs derived from *Haplophyllum tuberculatum* extract possessed broad-spectrum antibacterial activity against wastewater pathogens (e.g., *Escherichia coli* and *Staphylococcus aureus*) when synthesized with sodium borohydride, and facilitated the efficient catalytic degradation of dyes via intermediate organic molecules into environmentally benign products [13]. However, the high surface energy of AgNPs often leads to aggregation, compromising their catalytic performance. Therefore, researchers developed a loadable nanoparticle system, which solved this problem by fixing silver nanoparticles onto the substrate or the matrix of the composite material. Zhang et al. [14] fabricated magnetic Fe_3_O_4_@PDA nanocomposites loaded with AgNPs, which can serve as highly efficient catalysts for the reduction of organic pollutants in the presence of NaBH_4_. In the methylene blue (MB) reduction experiment, the Fe_3_O_4_@PDA-Ag composite completed the entire reduction reaction in only 7 min, achieving a 92.6% efficiency in converting MB to leuco-methylene blue (Leuco-MB). Moreover, the material maintains reusability after multiple cycles of MB reduction. Antibacterial tests against *Staphylococcus aureus*, *Bacillus cereus*, *Escherichia coli*, and *Pseudomonas aeruginosa* demonstrated that it possesses broad-spectrum antibacterial activity. Yang Rui’s team [15] first prepared magnetic Fe_3_O_4_ nanospheres by the "solvothermal method". Subsequently, according to the coordination principle, ultra-small (AgNPs) were adsorbed onto the surface of Fe_3_O_4_@PDA nanocomposites (FPN). Meanwhile, the inhibitory effects of Fe_3_O_4_@PDA@Ag nanocomposites (FAN) on Gram-negative bacterium *Escherichia coli* (*E. coli*), Gram-positive bacterium *Bacillus subtilis* (*B. subtilis*), and the drug-resistant strain *Salmonella typhi* (*S. typhi*) were investigated. The results showed that at a concentration of 200 μg/mL, the inhibition rates of FAN against *E. coli*, *B. subtilis*, and *S. typhi* reached as high as 99.9% at 60 min. FAN has excellent antibacterial performance and broad development prospects in the field of wastewater treatment. This method can enhance stability and reactivity for applications in water treatment, including dye degradation and antimicrobial processes [16,17,18,19].

Coal fly ash (CFA), a byproduct of coal-fired power plants, represents a critical industrial waste with a global annual production reaching millions of tons, driven by escalating energy demands [20]. The substantial accumulation and restricted recycling of CFA not only present considerable threats to the environment but also pose significant risks to human health. This urgent situation calls for the development and implementation of novel and innovative strategies to effectively utilize the resources at hand [21]. Composed primarily of SiO_2_ and Al_2_O_3_, with trace metal ions and amorphous phases, CFA offers notable advantages—a low cost, thermal stability, and superior surface and physicochemical properties—making it a viable silicon–aluminum raw material for zeolite synthesis and a versatile support for catalytic applications [22,23,24,25]. Its large surface area and ion exchange capacity render CFA an ideal substrate for immobilizing nanoparticles, enabling the fabrication of composites with retained antibacterial and catalytic properties for water purification [26,27]. Sun et al. [28] constructed composites with multiscale pore structures ranging from the nanometer to the macroscopic scale. The material exhibits a high interconnected porosity (up to 77.61%), suitable compressive strength (up to 23.79 MPa), and significant water permeation flux (549.86 m^3^·m^−2^·h^−1^ at 0.1 MPa). In addition, these composites have an excellent adsorption efficiency for Congo red (CR) in wastewater, reaching approximately 100%, with an adsorption capacity of 45.79 mg·g^−1^. This indicates that low-cost and environmentally friendly fly ash composites have potential application value in industrial-scale wastewater treatment. Wang et al. [29] prepared magnetic coagulants (Fe-AFA, Fe-BFA) by mixing acid-modified fly ash (AFA) and base-modified fly ash (BFA) with magnetic components, and used them as adsorbents for chemical oxygen demand (COD) in the desulfurization of wastewater. The results showed that Fe-AFA had the best COD adsorption performance and superparamagnetism, and the COD removal amount could reach 5.69 mg·g^−1^, which was 112.43% higher than that of raw fly ash. The reusability of the magnetic coagulants was studied. After four cycles of tests, the COD removal amount of Fe-AFA remained at 2.74 mg·g^−1^. This study provides a feasible scheme for the environmentally friendly utilization of fly ash as a low-cost adsorbent in wastewater treatment. Recent studies have demonstrated that CFA-based composites, such as alkaline/zinc-activated fly ash (A-FA/Zn) and fly ash cenosphere-supported photocatalysts (Ag-TON/FAC), exhibit efficient dye adsorption, photocatalytic degradation, and antibacterial activity [30,31,32]. However, the direct loading of AgNPs onto CFA for simultaneous microbial decontamination and dye degradation remains unexplored, presenting an unaddressed research gap.

Compared with nano-silver composite materials that utilize costly carriers such as graphene and mesoporous silica, CFA offers a significantly lower preparation cost, making it more suitable for large-scale applications. The incorporation of magnetic components, such as Fe_3_O_4_, enables the rapid separation and recycling of the material through an external magnetic field, effectively addressing the issues of secondary pollution and resource waste commonly associated with traditional nanomaterials (e.g., AgNPs), which are prone to loss due to their small particle size. Furthermore, the adsorption capacity of CFA (which physically adsorbs pollutants) synergizes with the chemical catalytic and antimicrobial properties of AgNPs, thereby boosting overall pollutant treatment efficiency. For instance, pollutants are first adsorbed onto the material’s surface, then efficiently degraded by AgNPs. Additionally, the rigid structure and surface functional groups of coal fly ash, including hydroxyl groups and silicon–oxygen bonds, act as robust anchoring sites for AgNPs. This not only minimizes Ag^+^ leaching and nanoparticle detachment but also ultimately extends the material’s service life.

In this study, we report the development of a novel AgNPs/magnetized coal fly ash (MCFA) composite via an impregnation–reduction–sintering route, aiming to integrate the antimicrobial and catalytic properties of AgNPs with the structural advantages of MCFA. The composite was comprehensively characterized to evaluate its structural, morphological, and physicochemical properties. Its antibacterial efficacy was evaluated against Gram-negative (*Escherichia coli*) and Gram-positive (*Staphylococcus aureus*) bacteria, while its catalytic performance was assessed via the degradation of methyl orange (MO), a model azo dye. Mechanistic insights into the antibacterial and catalytic processes were proposed based on the experimental results, offering a foundational framework for developing cost-effective, multifunctional wastewater treatment materials.

## 2. Results and Discussion

### 2.1. Characterization of AgNPs/MCFA

From the SEM diagram (Figure S1), it was evident that CFA exhibited a relatively uniform spherical shape and possessed a smooth surface. Acidification led to the removal of impurities from CFA, resulting in the formation of numerous new microstructures, thereby enhancing its ion exchange and adsorption capabilities. In addition, the magnetic particles synthesized by the co-precipitation method exhibit uniform growth on the surface of CFA and strong adhesion. The pretreatment with CFA facilitates the creation of a large number of anchoring sites for AgNPs, allowing for an even distribution of AgNPs across the pore surfaces of MCFA. The presence of small pores promoted controlled release mechanisms for nanoparticles, thereby enhancing their sustained antibacterial and catalytic efficacy [33]. 

The TEM analysis (Figure S2) confirmed the effectiveness of the loading method in achieving a uniform dispersion and controlled distribution of nanoparticles across the CFA surface. The small pore structure of the CFA served as a nanoreactor, spatially confining the nucleation sites during Ag reduction and enabling precise size regulation. Consequently, the synthesized AgNPs exhibited a narrow size distribution with an average diameter of 13.97 nm. High-resolution TEM (HRTEM) imaging revealed well-defined lattice fringes of AgNPs both deposited on and embedded within the CFA matrix. The measured interplanar spacing of 0.224 nm corresponded to the Ag (111) crystallographic plane but showed a contraction of 0.012 nm compared with the standard value (0.236 nm). This deviation suggested lattice compression, likely induced by interfacial Ag-O-Si bonding between the nanoparticles and the silica-rich CFA substrate.

The characterization of the AgNPs/MCFA composites was conducted using FT-IR spectroscopy, as illustrated in Figure S3. In contrast to CFA, MCFA displayed characteristic bands that indicate that the magnetization process did not introduce additional intensity bands; rather, it reflected a physical doping process without generating new functional groups. The broad absorption peaks at 3420 cm^−1^ and 3160 cm^−1^ are attributed to the strong stretching vibration of O-H bonds. Furthermore, the absorption peak at 1620 cm^−1^ is associated with the carboxyl group (-COO^−^). The hydroxyl group (-OH) in sodium citrate exhibited reductive properties, enabling the reduction of silver ions into nano-sized silver particles while simultaneously undergoing oxidation to form carboxyl compounds. Additionally, trisodium citrate can complex with silver ions to create a coating layer on the surface of AgNPs, thereby enhancing their dispersion and stability [34]. The peak at 1370 cm^−1^ was likely attributable to the symmetric stretching vibration of nitrate ions (NO_3_^−^) [35], further confirming the presence of NO_3_^−^ on the surface of AgNPs. At 1100 cm^−1^, the peak was attributed to the asymmetric stretching vibration of Si-O-Si bonds within CFA [36]. Following this, there was a noticeable decrease in intensity for this peak along with an emergence of a new Ag-O-Si peak at 470 cm^−1^ [37]. This observation suggested that the interaction between AgNPs and CFA involved more than mere physical adsorption; they also entailed chemical bond formation with the CFA surface.

The XRD analysis confirmed the presence of AgNPs/MCFA composites, as depicted in Figure S4. The graph clearly showed that a broad diffraction peak appears at approximately 25°, indicating a significant abundance of amorphous vitreous bodies within CFA [38]. These structural characteristics confer high hydrophilicity, surface activity, and adsorption properties to CFA. Following modification, a rightward shift was observed, signifying an increase in diffraction angle, a decrease in crystal plane spacing, and the introduction of lattice defects. Seven characteristic peaks corresponding to Fe_3_O_4_ nanoparticles (2θ = 30.3°, 35.5°, 43.3°, 53.4°, 57.3°, 63.8° and 74.2°) were identified in MCFA, which correspond to the (220), (311), (400), (422), (511), and (440) crystal faces based on the standard spectrum of Fe_3_O_4_. Additionally, four minor peaks at 38.1°, 44.5°, 64.3°, and 78.1° corresponding to the (111), (200), (220), and (311) crystal faces, respectively, were identified according to the standard spectrum for AgNPs with a face-centered cubic structure. In conclusion, it can be inferred from the XRD pattern that the CFA was magnetized and effectively loaded with AgNPs.

The VSM curve illustrated in Figure S5 demonstrates the remarkable superparamagnetism of AgNPs/MCFA, with a saturation magnetization of 68.7 emu g^−1^, which was 11.3 emu·g^−1^ lower than that of Fe_3_O_4_. This reduction can be attributed to the encapsulation effect of CFA, resulting in a weakening of the magnetic properties. In aqueous environments, AgNPs/MCFA tend to settle at the bottom when left undisturbed; however, upon introducing a magnet to the tube wall, a significant number of magnetic particles rapidly accumulated on the side with the magnet, facilitating in situ separation. Consequently, AgNPs/MCFA showed great potential for rapid recovery and repeated utilization in water treatment applications.

### 2.2. Effects of AgNO_3_ Concentration on Antibacterial Ability

**Figure 1:**
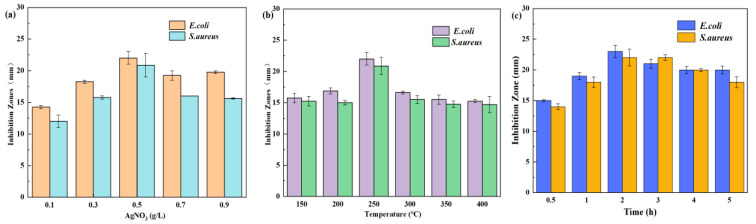
Effect of different AgNO_3_ concentrations (**a**), sintering temperature (**b**), and sintering time (**c**) on antibacterial properties.

The concentration of AgNO_3_ had a significant impact on the antibacterial properties of AgNPs, as demonstrated in Figure 1a. The antibacterial efficacy of AgNPs/MCFA against *E. coli* was more pronounced than that against *S. aureus*. This difference can be attributed to the greater susceptibility of the outer membrane of *E. coli* to disruption by AgNPs/MCFA, in contrast to the thick peptidoglycan layer present in *S. aureus*. Similar findings were reported by Waldo-Mendoza et al. in their study [39]. Furthermore, the antibacterial performance of the AgNPs/MCFA against both *E. coli* and *S. aureus* progressively improved with an increase in AgNO_3_ concentration from 0.1 to 0.5 g·L^−1^. However, during the reduction reaction at elevated concentrations of AgNO_3_, a substantial number of nuclei for AgNPs were formed, leading to localized supersaturation conditions. This phenomenon promoted the formation of numerous small particles and resulted in an uneven distribution of AgNPs on the surface of MCFA, thereby diminishing its antimicrobial efficacy. Consequently, it was determined that the optimal concentration of AgNO_3_ for synthesizing AgNPs on the surface of MCFA was 0.5 g·L^−1^.

### 2.3. Effect of Sintering Temperature and Sintering Time on Antibacterial Ability

The impact of high-temperature sintering and sintering time on the antibacterial efficacy of the AgNPs/MCFA was examined, with the results illustrated in Figure 1b,c. High-temperature sintering facilitated a more secure anchoring of AgNPs onto the surface of MCFA, thereby preventing peeling or scattering during application and enhancing long-term performance. The thermal sintering process promoted inter-particle bonding among silver powder particles, leading to a densely interconnected structure. An optimal combination of sintering temperature and time can enhance the crystallization and stability of AgNPs, further optimizing their antibacterial properties. At a sintering temperature of 250 °C for a duration of 2 h, the AgNPs/MCFA exhibited superior antibacterial effectiveness against *E. coli* and *S. aureus*, demonstrating inhibition zones measuring 23 mm and 22 mm, respectively. However, excessively high temperatures coupled with prolonged exposure may lead to the oxidation of AgNPs [40], consequently reducing their antibacterial efficacy.

### 2.4. The MIC of AgNPs/MCFA

The minimum inhibitory concentration (MIC) is defined as the lowest concentration of an antimicrobial agent that prevents the visible growth of a microorganism when cultured at a specific temperature for 18 h. A lower MIC value indicated the greater susceptibility of the microorganism to the antimicrobial agent. Therefore, the antibacterial property was enhanced [41]. As shown in Table 1, when the concentration of AgNPs/MCFA was equal to or exceeded 2 mg·mL^−1^, it demonstrated inhibitory effects on both *E. coli* and *S. aureus*, as evidenced by the clarification of the bacterial suspension in the test tube. The MIC values for *E. coli* and *S. aureus* were determined to be 0.5 mg·mL^−1^ and 2 mg·mL^−1^, respectively. These findings indicated that AgNPs/MCFA can effectively suppress the growth of both bacteria in aqueous solution. Notably, a higher concentration of AgNPs/MCFA was required to inhibit *S. aureus* compared with *E. coli*, suggesting a stronger antibacterial efficacy against *E. coli*. This observation aligned with the results obtained from experiments assessing the antibacterial zone.

### 2.5. Impact of AgNPs/MCFA on Bacterial Nucleic Acid Leakage, Protein Leakage, and Electrical Conductivity

**Figure 2:**
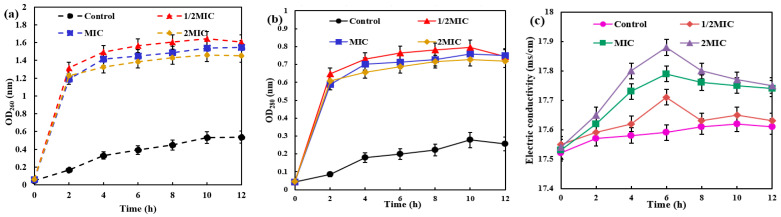

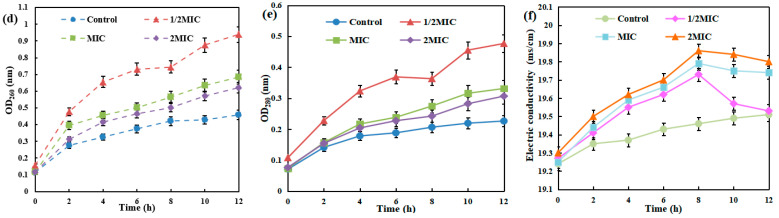
Nucleic acid, protein, and electrolyte leakage from *E. coli* (**a**–**c**) and *S. aureus* (**d**–**f**).

The disruption of the bacterial cell membrane is accompanied by the leakage of intracellular contents. The impact of AgNPs/MCFA on this leakage can be quantified by measuring the optical density or specific components present in the supernatant of the bacterial culture medium. As shown in Figure 2, AgNPs/MCFA exerted varying effects on the membrane integrity of *E. coli* and *S. aureus*, with distinct levels of impact observed. From the perspective of protein leakage, it was noted that the soluble protein content progressively increased with extended culture time. Moreover, an increase in AgNPs/MCFA concentration resulted in a significant elevation in soluble protein levels compared with those observed in the control group. Remarkably, AgNPs/MCFA showed a greater destructive effect on the cell membrane of *E. coli*, leading to a rapid rise in soluble protein levels during the final 2 h of culture. Ultimately, when the concentration of AgNPs/MCFA was 1/2 MIC, the destructive effects on the cell membranes of both *E. coli* and *S*. *aureus* were enhanced. This destructive effect resulted in the increased leakage of soluble proteins. In terms of nucleic acid leakage, its trend over time closely mirrored that observed for protein leakage as culture duration extended.

After bacterial lysis, the release of intracellular K^+^, Ca^2+^, and other ions into the extracellular environment can be monitored [42]. This phenomenon can be quantified by measuring electrical conductivity, thus enabling an assessment of the extent of membrane damage. The electrical conductivity in the control group showed minimal variation. As the concentration of AgNPs/MCFA increased, cell membrane damage intensified, leading to a corresponding rise in measured conductivity. Over time, the conductivity of *E. coli* and *S. aureus* initially increased and then decreased, reaching peak values at 6 and 8 h, respectively. The initial increase in conductivity may be attributed to cell membrane damage leading to cell leakage. However, as time progressed, the rate of charge flow within the bacteriostatic solution diminished, ultimately culminating in the destruction of the cell membrane and a subsequent decline in conductivity.

### 2.6. Antimicrobial Kinetics

**Figure 3:**
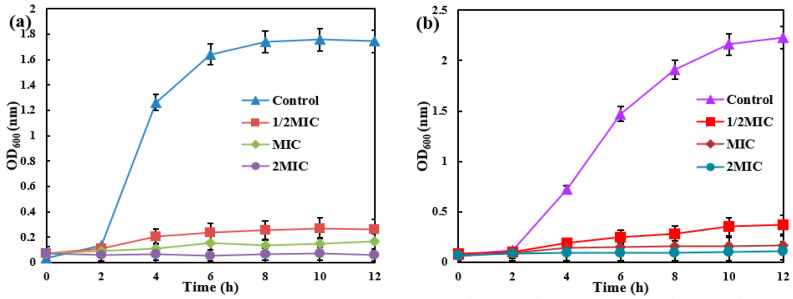
The growth of *E. coli* (**a**) and *S. aureus* (**b**) under different MIC of AgNPs/MCFA.

Different amounts of AgNPs/MCFA were introduced into culture media inoculated with *E. coli* and *S. aureus*, as shown in Figure 3. The optical density (OD) at 600 nm (OD_600_) was measured to monitor bacterial growth, where higher values indicate microbial density. The results revealed a concentration-dependent reduction in OD_600_ with escalating levels of AgNPs/MCFA, suggesting a pronounced inhibitory effect on bacterial proliferation. The studies confirmed that AgNPs/MCFA effectively suppresses the growth of both *E. coli* (Gram-negative) and *S. aureus* (Gram-positive), without significant differences in efficacy between the two bacterial types. This broad-spectrum activity may arise from AgNPs/MCFA targeting common bacterial structures, such as cell membranes or energy metabolism, rather than strain-specific components. These findings underscore the potential of AgNPs/MCFA as an effective antibacterial agent in aqueous environments, thereby supporting its development for nano-silver-based aquatic antimicrobial applications.

### 2.7. Catalytic Performance of AgNPs/MCFA for Degradation of MO

**Figure 4:**
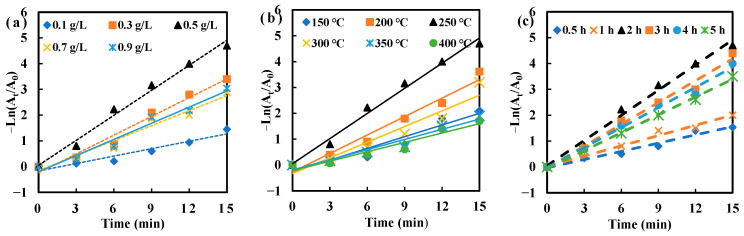
Kinetic model of MO degradation under different preparation conditions (AgNO_3_ concentration (**a**), sintering temperature (**b**), and sintering time (**c**)).

Figure 4 depicted the kinetic fitting curve for MO degradation catalyzed by AgNPs/MCFA produced at different AgNO_3_ concentrations, sintering temperatures, and sintering times. The results showed that MO degradation followed first-order kinetics, as evidenced by the strong linear correlation observed in the kinetic fitting curves. At lower AgNPs concentrations, insufficient synthesis led to a limited number of catalytically active sites, resulting in a reduced efficiency in MO degradation due to insufficient electron mediation [43]. Conversely, the overproduction of AgNPs can lead to aggregation and the formation of larger particles, thereby reducing the effective catalytic surface area and obstructing active sites, ultimately reducing catalytic efficiency.

Furthermore, the accessible surface area was limited to lower sintering temperatures due to the underdeveloped pore structure. As a result, hydroxyl groups (-OH) were not properly exposed, diminishing the number of binding sites for AgNPs and slowing down the kinetics of MO degradation. Conversely, high-temperature sintering posed a risk of pore collapse, leading to a reduction in total surface area. This phenomenon significantly decreased the interaction between CFA and AgNPs by promoting the consolidation of AgNPs into larger particles through processes such as Ostwald ripening or coalescence [44]. Such aggregation reduced the number of active sites available and disrupted their uniform distribution.

Similarly, the duration of sintering was crucial. Insufficient sintering time failed to completely remove residual carbon and water from CFA, leading to a reduced specific surface area and the incomplete exposure of hydroxyl groups (Si-OH) as well as defect sites in silica–aluminum oxides. Appropriately extending the sintering time enhanced the crystallinity of AgNPs, promoting an orderly atomic arrangement and enhancing both the thermal and chemical stability of the material. However, excessive sintering time resulted in pronounced grain growth, diminishing surface activity.

By systematically optimizing the sintering temperature and duration, the synergistic interaction between CFA and AgNPs, termed "carrier-active site", can be maximized, thereby significantly enhancing the catalytic degradation efficiency of MO. Consequently, the highest rate constant *k_a_* value was achieved for AgNPs/MCFA prepared under conditions of 0.5 g·L^−1^ AgNO_3_ concentration, 250 °C, and a duration of 2 h (Table S1), indicating the optimal catalytic degradation rate of MO. To quantify this effect, we introduced a normalized activity parameter defined as the ratio of rate constant *k_a_* to the total mass of the catalyst (m), k′ (k′ = k/m). The catalytic activity parameter for AgNPs/MCFA in reducing MO was obtained to be 6.2 min^−1^·g^−1^. Therefore, AgNPs/MCFA served as an effective catalyst for degrading organic dyes and holds significant potential for application in wastewater treatment.

### 2.8. Reusability of AgNPs/MCFA

**Figure 5:**
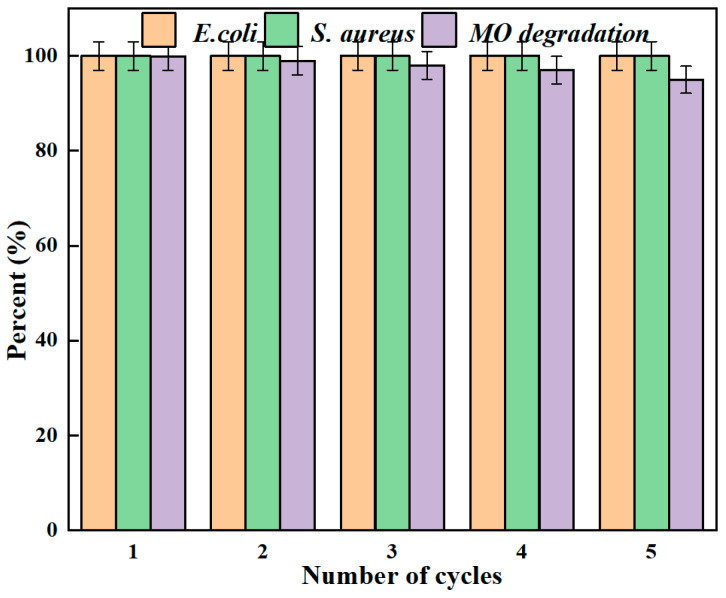
Antibacterial and MO degradation of AgNPs/MCFA recycling for 5 times.

Reusability is a critical indicator of material quality. The capacity to maintain high antimicrobial efficacy and degradation rates after undergoing multiple testing cycles contributed significantly to reducing application costs. Furthermore, to eliminate impurities, the particles were sequentially treated with alcohol and water. After being used continuously for five cycles, they still exhibited commendable antibacterial properties and degradability, as shown in Figure 5. The small and uniformly dispersed silver nanoparticles maximize the surface contact with the CFA matrix, while the strong Ag-O-Si chemical bonds firmly fix these nanoparticles onto the silicate framework of the CFA, minimizing the leaching of silver ions and the shedding of particles even under repeated use and mechanical stress. AgNPs/MCFA can be employed to eliminate harmful microorganisms and toxic contaminants from water. However, its recyclability and reusability in water treatment processes were of critical importance. A notable advantage of AgNPs/MCFA over other nanoparticles lies in their ability to be easily separated from water by applying an external magnetic field. This facilitated the recovery of particles following each treatment cycle, thus minimizing the potential release of nanoparticles into the environment.

### 2.9. Proposed Antibacterial and Degradation Mechanism of AgNPs/MCFA

**Figure 6:**
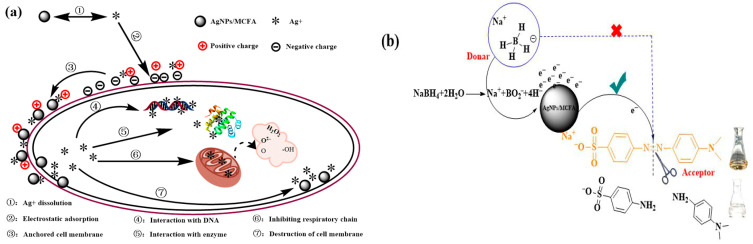
Mechanism of AgNPs/MCFA-induced damage to bacterial cells (**a**) and reductive degradation of MO (**b**).

After acid treatment, the surface morphology of CFA transitions from a smooth to a rough texture, resulting in an increase in porosity, specific surface area, and physical adsorption capacity. This micrometer-scale pore structure facilitated bacterial adsorption and enhanced accessibility for AgNPs, ultimately leading to damage to microbial cells. AgNPs inherently possessed a positive charge, which enabled effective electrostatic adsorption with negatively charged bacterial cell walls. Upon disrupting these cell walls and membranes, AgNPs directly interact with intracellular DNA. Through specific interactions with the outer-ring nitrogen atoms in bases such as adenine and pyrimidine, they compromise the normal structure and physiological function of DNA. Furthermore, AgNPs can engage with and inactivate disulfide bonds and sulfhydryl groups within enzymes responsible for cellular metabolism, particularly respiratory enzymes, thereby generating reactive oxygen species (ROS) that induce oxidative stress in microorganisms. This process results in the loss of active intracellular electrolytes (e.g., K^+^, Ca^2+^, Cl^−^, and PO_4_^3−^), nucleic acids, and proteins. These components were subsequently released into extracellular solutions (Figure 6a). Consequently, the measurements of solution conductivity (ion leakage), along with OD_260_ (nucleic acid release) and OD_280_ (protein release) absorbance, in cultures treated with varying MIC levels of AgNPs/MCFA, provide robust confirmation of membrane disruption and cellular content leakage. Moreover, Gram-positive bacteria (e.g., *S. aureus*) exhibit a significantly higher resistance to AgNPs compared with Gram-negative bacteria (e.g., *E. coli*). This disparity in susceptibility can be attributed to fundamental differences in cell envelope structure. Specifically, the dense peptidoglycan layer in Gram-positive organisms may limit nanoparticle penetration and subsequent intracellular damage.

After acid treatment, the surface properties of CFA not only improved but also exhibited a positive charge characteristic. This facilitated strong electrostatic adsorption with dye anions in water, thereby enhancing the adsorption performance of CFA. Although NaBH_4_ was a powerful reducing agent, its aqueous solution failed to effectively reduce MO due to the substantial difference in redox potential between MO and NaBH_4_. Consequently, these reactions were kinetically hindered despite being thermodynamically feasible. The interaction between MO and NaBH_4_ can be classified as an electron transfer process, wherein NaBH_4_ served as the electron donor while MO functioned as the electron acceptor [45]. AgNPs/MCFA served as an effective intermediary for the H^−^ ions released during the interaction between MO and NaBH_4_, thus promoting efficient electron transfer. The presence of AgNPs accelerated the reduction reactions owing to their large surface area. Before reduction, both reactants were adsorbed onto the surface of AgNPs/MCFA. The surface characteristics of acid-treated CFA were enhanced with positively charged properties. These properties facilitated this process by attracting reactants to the AgNPs/MCFA surface through electrostatic interactions. Subsequently, H^−^ ions transferred electrons to the AgNPs/MCFA surface, enabling MO molecules to accept electrons. This weakened the azo double bond (-N=N-), thereby driving the color degradation reaction (Figure 6b).

## 3. Materials and Methods

### 3.1. Chemicals and Bacteria

Coal fly ash (CFA, 5000 mesh) was procured from Henan Borun Casting Material Co., Ltd. (Zhengzhou, China). Sodium borohydride was obtained from Shanghai Lingfeng Chemical Reagent Co., Ltd. (Shanghai, China). MO, AgNO_3_, FeCl_3_, FeSO_4_, and sodium citrate (C_6_H_5_Na_3_O_7_) were sourced from Sinopharm Group Chemical Reagent Co., Ltd. (Shanghai, China). Peptone, yeast extract, agar, and NaCl were acquired from Fuchen Chemical Reagent Co., Ltd. (Tianjin, China). *Escherichia coli* (*E. coli*) was selected as a representative strain of Gram-negative bacteria, and *Staphylococcus aureus* (*S. aureus*) as a representative strain of Gram-positive bacteria. *Escherichia coli* (ATCC 25922) and *Staphylococcus aureus* (ATCC 6538) were procured from the China Center of Industrial Culture Collection (Wuhan, China).

### 3.2. Preparation of Composite Materials

#### 3.2.1. Magnetization of CFA

The CFA was stirred in a 10 wt% HCl solution at 90 °C for 1 h, followed by filtration and washing to neutral pH, and then dried to obtain acidified CFA. Subsequently, a mixed solution was prepared by combining equal volumes of 0.012 mol·L^−1^ FeCl_3_ with 0.005 mol·L^−1^ FeSO_4_. The acidified CFA was introduced into the mixed solution, which was then heated to 70 °C on a magnetic stirrer, and the pH of the solution was adjusted to 8–9 using 1 mol·L^−1^ NaOH. The beaker was then subjected to ultrasonic treatment for 30 min to achieve ultrasonic dispersion. Following this, the mixture was aged for an additional hour. Finally, the precipitate was filtered out, washed until it reached a neutral pH, and dried to obtain magnetic CFA (MCFA).

#### 3.2.2. Preparation of AgNPs/MCFA

The MCFA was immersed in AgNO_3_ solutions of varying concentrations (0.1, 0.3, 0.5, 0.7, and 0.9 g·L^−1^) under dark conditions with continuous stirring for 2 h. After that, an appropriate amount of sodium citrate was added as a reducing agent, and the mixture was stirred for 24 h to promote the formation of AgNPs. Finally, the AgNPs-loaded MCFA was filtered, washed, and dried. The dried sample was sintered in a tube furnace under an inert atmosphere with a controlled heating rate of 10 °C·min^−1^ to specific sintering temperatures (150, 200, 250, 300, 350, and 400 °C), with a sintering time (0.5, 1, 2, 3, 4, and 5 h) maintained throughout the process. After allowing the sample to cool naturally, it was ground to obtain the final product, AgNPs/MCFA.

### 3.3. Characterization of Materials

Scanning electron microscopy (SEM, Hitachi Regulus 8100, Tokyo, Japan) and transmission electron microscopy (TEM, FEI Talos F200X, Hillsboro, OR, USA) were used to characterize the surface morphology and microstructure of the materials. Fourier transform infrared spectroscopy (FT-IR, Thermo Fisher Scientific, Waltham, MA, USA) was employed to investigate the functional groups and chemical bonds in the range of 500 cm^−1^ to 4000 cm^−1^. X-ray diffraction (XRD, Smart Lab SE, Tokyo, Japan) was utilized to characterize the mineralogical composition and phase structure. Value Stream Mapping (VSM, Lakeshore 7404, Carson, CA, USA) was used to obtain the saturation magnetization of the material.

### 3.4. Determination of the Antibacterial Activity of AgNPs/MCFA

#### 3.4.1. Size of the Antibacterial Zone

Typically, bacteria exhibit uniform growth on agar media. Nevertheless, in the presence of antimicrobial materials, the antimicrobial components diffuse into specific regions of the medium, impeding bacterial growth and creating an inhibition zone. Notably, the larger the relative area of this inhibition zone, the more potent the antibacterial effect. The colonies of *E. coli* and *S. aureus* were selected and cultivated in LB liquid medium (comprising 10 g·L^−1^ peptone, 5 g·L^−1^ yeast extract, and 10 g·L^−1^ sodium chloride) at 37 °C. Upon reaching a measured concentration of viable bacteria at 1.0 × 10^8^ CFU·mL^−1^, 0.1 mL of *E. coli* and *S. aureus* was separately inoculated onto LB solid medium (containing 10 g·L^−1^ peptone, 5 g·L^−1^ yeast extract, 10 g·L^−1^ NaCl, and 20 g·L^−1^ agar). After flame sterilization, a 9.0 mm round hole was drilled in the plate with a hole puncher, and then 0.1 g of the aforementioned AgNPs/MCFA composite was filled into the hole and finally cultured in a 37 °C incubator for 18 h. The diameter of the resulting antibacterial zone for each sample was then measured in millimeters to assess its effectiveness against the respective bacterial strains present on the plate.

#### 3.4.2. Minimum Inhibitory Concentration (MIC)

The MIC of the AgNPs/MCFA composite was determined using the double gradient dilution method [46], with sterile distilled water employed for the gradient dilution. *E. coli* and *S. aureus* were cultured to the logarithmic phase and diluted into the LB medium at a concentration of 1.0 × 10^8^ CFU·mL^−1^. Specifically, the bacterial solution (1.0 × 10^8^ CFU·mL^−1^) was added to each sterile centrifuge tube numbered from 1 to 9. Each tube received 1 mL of the bacterial solution, except for tube 1, which was filled with 1.8 mL. Subsequently, 0.2 mL of the AgNPs/MCFA composite suspension was added to the initial tube, thoroughly mixed, and then transferred by dilution in increments of 1 mL from one tube to the next in sequence until reaching the ninth tube. Finally, 1 mL of the solution was discarded from the ninth tube. All samples were placed in a thermostatic oscillator and incubated at 37 °C for 18 h. The MIC value was defined as the lowest concentration at which no turbidity was visible upon visual inspection.

#### 3.4.3. Nucleic Acid, Protein Leakage, and Electrical Conductivity

*E. coli* and *S. aureus* were cultured to a concentration of 1.0 × 10^8^ CFU·mL^−1^. The bacterial cells were harvested by centrifugation at 5000 rpm for 30 min. After centrifugation, the cells were washed twice with normal saline. The washed cell pellets were resuspended in saline until the optical density at a wavelength of 600 nm (OD_600_) of the suspension reached 0.6. The bacterial suspensions were then treated with the AgNPs/MCFA composite at concentrations equivalent to 2 times the minimum inhibitory concentration (2 MIC), the minimum inhibitory concentration (MIC), and half of the minimum inhibitory concentration (0.5 MIC). These mixtures were incubated in a thermostatic shaker at 37 °C for 12 h. During the incubation process, 2 mL aliquots were taken out every 2 h for analysis. The absorbance of the bacterial suspensions was measured at 260 nm (nucleic acid release) and 280 nm (protein release), in order to evaluate the cellular leakage. The electrical conductivity (ion leakage) of the suspensions was determined using a conductivity meter (Shanghai Yi Dian Scientific Instruments Co., Ltd., Shanghai, China). For all the measurements, bacterial suspensions that did not contain the AgNPs/MCFA composite were used as blank controls.

#### 3.4.4. Determination of Antimicrobial Kinetics

*E. coli* and *S. aureus* were cultured to a concentration of 1.0 × 10^8^ CFU·mL^−1^, and 1.0 mL of each bacterial suspension was inoculated into 100 mL of LB medium. The AgNPs/MCFA composite, which had been diluted with sterile water, was introduced at final concentrations corresponding to 2 MIC, MIC, and 0.5 MIC. The cultures were then incubated in a thermostatic shaker at 37 °C for 12 h. During the incubation period, 2 mL aliquots were taken out every 2 h, and the absorbance at 600 nm was measured using a UV–vis spectrophotometer (Shanghai Yi Electric Analytical Instrument Co., Ltd., Shanghai, China) to plot the antibacterial kinetics curve. A control group, devoid of any AgNPs/MCFA composite, was maintained under identical conditions for comparison.

### 3.5. The Catalytic Efficiency of AgNPs/MCFA

The catalytic activity of the AgNPs/MCFA composite was assessed in the reduction reaction of MO, which served as a model dye, with NaBH_4_ acting as the reducing agent. In a typical experiment, 100 mL of MO (100 mg·L^−1^) and 2 mL of NaBH_4_ (0.2 mol·L^−1^) were combined in a 250 mL conical flask, and 50 mg of the AgNPs/MCFA composite was placed on a magnetic stirrer at 30 °C. At specific time intervals, 2 mL of the reaction mixture was taken out. Subsequently, UV–visible spectrophotometry was used to monitor the degradation of MO at its maximum absorbance peak (OD_465_). After the reaction had proceeded for 15 min, the degradation rate (*R*) was calculated using the following formula:

(1) $$R\left(\%\right)=\frac{C_{0}\;-\;C_{t}}{C_{0}}\times 100$$

where *C*_0_ and *C_t_* indicate the mass concentration of the dye at the beginning and *t*, respectively (mg·L^−1^).

Generally, the reduction reaction can be assumed as a pseudo-first-order kinetics reaction due to the excess concentration of NaBH_4_ compared with MO. It can be described by the Langmuir–Hinshelwood equation [47].

(2) $$ln(\frac{A_{t}}{A_{0}})={-k}_{a}t\;$$

where *A*_0_ and *A*_t_ indicate the absorbance value of the dye at the beginning and *t*, respectively; *k_a_* is the reaction rate constant (min^−1^).

### 3.6. Reusability of AgNPs/MCFA

The reusability of the AgNPs/MCFA composite is a crucial parameter for evaluating its performance [48]. After each application, the AgNPs/MCFA composite was retrieved and thoroughly washed with alcohol and deionized water, dried, and then utilized for subsequent antibacterial and MO dye degradation experiments. The bacteriostatic rate and dye degradation rate were employed to evaluate the efficacy of each experiment.

The number of colonies in the original bacterial solution and the number of colonies after inhibition were determined by the plate counting method. Based on the data obtained, the antibacterial rate of the AgNPs/MCFA composite was calculated according to the following formula:

(3) $$Antibacterial\;rate\left(\%\right)=\frac{N_{0}\;-\;N}{N_{0}}\times 100$$

where *N*_0_ is the initial colony number and *N* is the number of colonies after inhibition.

The reusability experiment of the AgNPs/MCFA composite in dye degradation was conducted following the same procedure as described in Section 3.5.

## 4. Conclusions

In summary, this study successfully developed a magnetic AgNPs/MCFA composite with multifunctional performance for wastewater treatment. In terms of catalytic degradation, the composite achieved a 99.89% degradation of methyl orange (MO) within 15 min. For antimicrobial activity, it exhibited strong inhibitory effects against both Gram-negative *E. coli* (MIC = 0.5 mg·mL^−1^) and Gram-positive *S. aureus* (MIC = 2 mg·mL^−1^). The composite could be recovered with a magnet, and it retained over 90% of its catalytic and antimicrobial activity after five cycles of reuse, significantly reducing operational costs. From an industrial perspective, this composite addresses the high cost of traditional AgNP materials (via the utilization of waste CFA) and the difficulty in recycling nanocatalysts (via magnetic separation). The composite material was synthesized through a simple one-pot method without the use of toxic reagents, making it suitable for large-scale production and providing an economical, efficient, and environmentally friendly solution for comprehensive wastewater treatment (simultaneously removing dyes and pathogens). This aligns with the industrial demand for “low-cost, high-efficiency and sustainable” water treatment technologies, especially for small to medium-sized sewage treatment plants with limited budgets. However, future research should further evaluate the performance of this composite material in complex actual wastewater environments containing high concentrations of ions, organic matter, or heavy metals, as well as the potential interference effects caused by coexisting substances.

## Figures and Tables

**Table 1:** Determination of the MIC value of AgNPs/MCFA.

No.	Concentration (mg·mL^−1^)	Tested Bacteria	
		*E. coli*	*S. aureus*
1	64	−	−
2	32	−	−
3	16	−	−
4	8	−	−
5	4	−	−
6	2	−	−
7	1	−	+
8	0.5	−	+
9	0.25	+	+

Note: “+” result signifies that the test tube exhibits turbidity with visible bacterial growth; “−” result indicates that the test tube is clear with no observable bacterial growth.

## Data Availability

All data supporting the findings of this study are available within the paper.

## Supplementary Materials

The following supporting information can be downloaded at: https://www.mdpi.com/article/10.3390/molecules30153155/s1, Figure S1: SEM image of CFA (a), MCFA (b), and AgNPs/MCFA (c); Figure S2: TEM image of AgNPs/MCFA; Figure S3: FTIR spectrum of CFA (a), MCFA (b), and AgNPs/MCFA (c); Figure S4: XRD pattern of CFA (a), MCFA (b), and AgNPs/MCFA (c); Figure S5: VSM image of Fe_3_O_4_ (a), AgNPs/MCFA (b); Table S1: Parameters and regression coefficients for the kinetic model and degradation rate.

## Abbreviations

The following abbreviations are used in this manuscript:

MCFA	magnetized coal fly ash
AgNPs	silver nanoparticles
MO	methyl orange
CFA	coal fly ash
MIC	minimum inhibitory concentration
ROS	reactive oxygen species
