# Supplementary material for: Dual-Functional AgNPs/Magnetic Coal Fly Ash Composite for Wastewater Disinfection and Azo Dye Removal
Source: Molecules. 2025 Jul 28;30(15):3155. doi: 10.3390/molecules30153155 (PMC12348525; doi:10.3390/molecules30153155)
Supplement: Supplementary file 1 [file molecules-30-03155-s001.zip › molecules-3768736-supplementary.pdf]

Supplementary Materials

Dual-Functional AgNPs/Magnetic Coal Fly Ash Composite for Wastewater Disinfection and Azo Dye Removal

Lei Gong [1,2,*], Jiaxin Li [2], Rui Jin [2], Menghao Li [3], Jiajie Peng [3] and Jie Zhu [1,*]

1 National-Local Joint Engineering Research Center of Biomass Refining and High-Quality Utilization, Institute of Urban & Rural Mining, Changzhou University, Changzhou 213164, China
2 School of Petrochemical Engineering, Changzhou University, Changzhou 213164, China
3 School of Environment Science and Engineering, Changzhou University, Changzhou 213164, China
* Correspondence: gonglei@cczu.edu.cn (L.G.); zhujie@cczu.edu.cn (J.Z.)

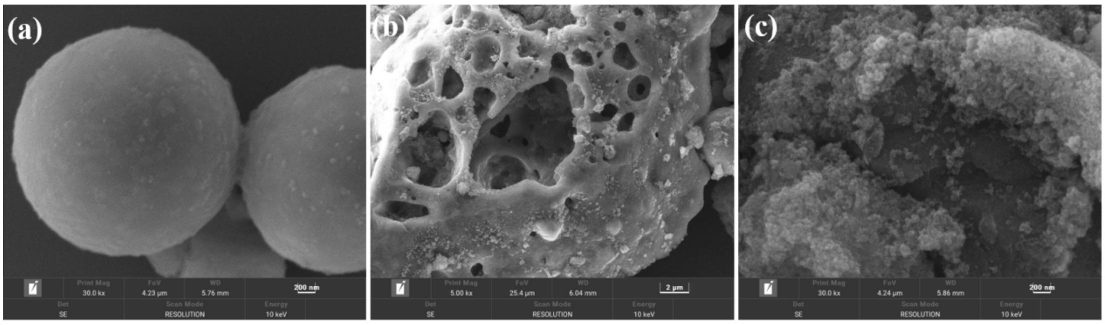

Figure S1. The SEM image of CFA (a), MCFA (b), and AgNPs/MCFA (c)

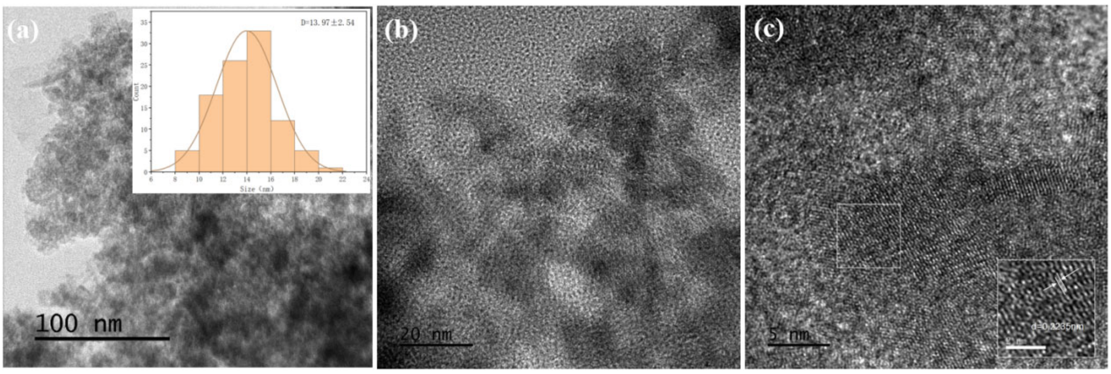

Figure S2. The TEM image of AgNPs/MCFA

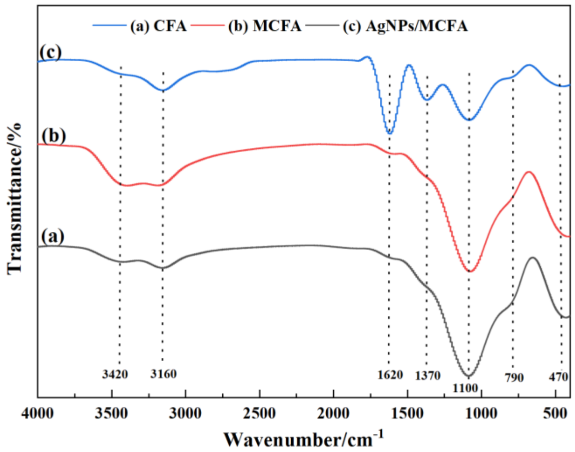

Figure S3. The FTIR spectrum of CFA (a), MCFA (b), and AgNPs/MCFA (c)

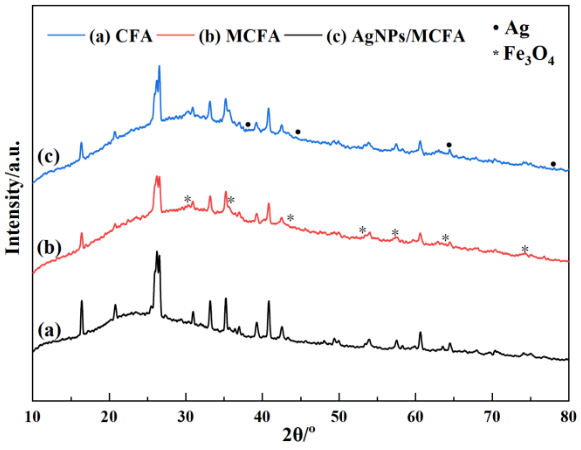

Figure S4. The XRD pattern of CFA (a), MCFA (b), and AgNPs/MCFA (c)

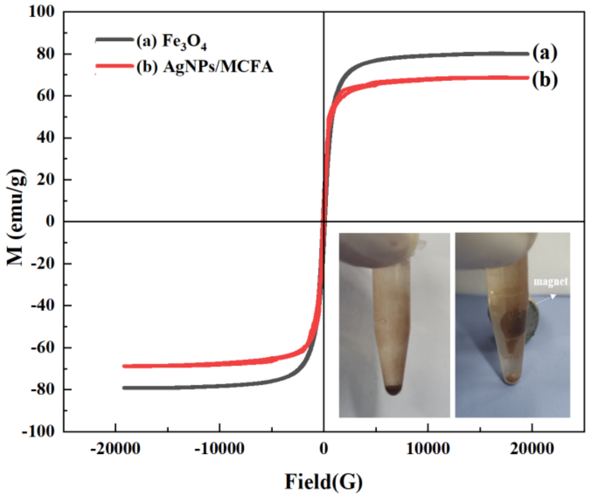

Figure S5. The VSM image of Fe_3O_4 (a), AgNPs/MCFA (b)

Table S1. Parameters and regression coefficients for the kinetic model and degradation rate

**Conditions**	k_a **(min^{-1})**	R**%**	R^2
0.1 g/L	0.0967	30.56	0.9297
0.3 g/L	0.2267	65.48	0.9775
0.5 g/L	0.3133	99.89	0.9887
0.7 g/L	0.1913	75.34	0.9624
0.9 g/L	0.2027	76.88	0.9697
150 ℃	0.1407	39.72	0.9107
200 ℃	0.2413	54.64	0.9703
250 ℃	0.3133	99.89	0.9887
300 ℃	0.2133	60.98	0.9373
350 ℃	0.1267	55.06	0.9379
400 ℃	0.1133	50.92	0.9310
0.5 h	0.1023	40.23	0.9674
1 h	0.1333	67.56	0.9828
2 h	0.3133	99.89	0.9887
3 h	0.2933	86.75	0.9848
4 h	0.2667	83.55	0.9889
5 h	0.2333	75.28	0.9969
